# Changes in coagulation functions and hemorheological parameters may predict hematoma formation after total knee arthroplasty

**DOI:** 10.1186/s13018-016-0370-5

**Published:** 2016-03-24

**Authors:** Ning Liu, Simin Luo, Cheanglek Hang, Zhengang Zha, Jieruo Li, Wenrui Wu, Dabiao Hou

**Affiliations:** The First Affiliated Hospital, Jinan University, Guangzhou, 510632 China; Institute of Orthopaedic Disease Research, Jinan University, Guangzhou, 510632 China; Macau Medical Science & Technology Research Association, Macau, China

**Keywords:** Activated partial thromboplastin time, Prothrombin time, Total knee arthroplasty, Hematoma, Apixaban

## Abstract

**Background:**

Hematoma formation around the knee is commonly seen after total knee arthroplasty (TKA) and may cause patient discomfort and worry regarding the success of the surgery. This study aimed to evaluate the coagulation functions and hemorheological parameters in patients undergoing TKA and investigate their associations with hematoma formation.

**Methods:**

This study prospectively included 146 patients treated for knee osteoarthritis by unilateral TKA between August 2013 and August 2014. Apixaban was administered twice during the 12–24-h period after surgery. Blood coagulation functions were evaluated according to activated partial thromboplastin time (APTT), prothrombin time (PT), thrombin time, and fibrinogen preoperatively and on postoperative days 1, 3, 7, and 14. Hemorheological parameters were also measured. Patients were divided into a hematoma group and a non-hematoma group for comparison.

**Results:**

On postoperative day 1, the hematoma group showed significantly prolonged APTT and PT and significantly decreased hematocrit relative to baseline values (*P* < 0.05). The whole blood high shear rate, whole blood low shear rate, plasma viscosity, and hematocrit did not differ significantly between the two groups at baseline or from postoperative days 1–14 in (*P* > 0.05).

**Conclusions:**

Prolonged APTT and PT on the first day after TKA as well as decreased hematocrit may indicate an increased risk of hematoma formation. Postoperative use of apixaban may promote the formation of ecchymoses but is not a major contributing factor.

## Background

Total knee arthroplasty (TKA) is an effective treatment for advanced pathological conditions of the knee. TKA is performed increasingly often due to the global aging trend. However, this procedure is associated with some complications. Patients undergoing TKA are often at a hypercoagulable state and prone to the development of thrombosis. If not properly managed, the incidence of venous thromboembolism (VTE) in these patients can be as high as 40–80 %, resulting in a mortality rate of 2 % due to symptomatic pulmonary embolism [1, 2].

The American Academy of Orthopaedic Surgeons and the American College of Chest Physicians developed new evidence-based guidelines for VTE prophylaxis after total joint arthroplasty in 2013. According to the updated guidelines, one of the following agents should be used for a minimum of 14 days after surgery: warfarin, low-molecular-weight heparin, fondaparinux, aspirin, rivaroxaban, dabigatran, apixaban, or portable mechanical compression. Moreover, bleeding tendencies in these patients must be monitored carefully [2]. Although these prophylaxis treatments very rarely cause major hemorrhaging, their use during the first 2 weeks after TKA more commonly contributes to the occurrence of ecchymoses and limb swelling, complications that may cause worry among patients and delay the initiation of exercise, leading to compromised clinical outcomes [3, 4].

Many factors have been proposed to be related to hematoma formation after TKA, such as the concurrent use of continuous femoral nerve block and an anticoagulant [3], postoperative drainage patterns [4–7], tourniquet use during operation [8, 9], postoperative anticoagulant use [10], and postoperative lower limb positioning [11, 12]. However, the specific underlying reasons for hematoma formation after TKA remain unclear. We hypothesized that hypercoagulability and hemorheological changes are involved in the development of ecchymoses after TKA. To test this hypothesis, in this study, we evaluated the coagulation functions and hemorheological parameters during the perioperative period of TKA and analyzed potential associations with hematoma formation.

## Methods

### Patients

This study included 146 patients with knee osteoarthritis who were treated between August 2013 and August 2014 at our hospital. The patients included 51 males and 95 females and had a mean age of 70 years (range, 64–78 years). Osteoarthritis was diagnosed according to the criteria provided by the American College of Rheumatology in 1995 [13]. Only patients with surgical indications for TKA were included in this study. The surgical indications for TKA were several instability, pain, deformity, and dysfunction of the knee joint. All patients underwent unilateral TKA. Exclusion criteria were the patients with end-stage renal disease, severe liver dysfunction, major comorbidities (bleeding disorders, ischemic heart disease, and peripheral vascular diseases), and any kind of cancer disease. Informed written consent was obtained from all patients, and the study was approved by the Ethics Committee of the First Affiliated Hospital, Jinan University.

### Postoperative management

Drainage tubes were placed postoperatively and removed within 24–48 h. The tubes were not temporarily clipped after TKA. Antibiotics were used for 3 days to prevent infection. Patients were instructed to wear elastic stockings. Apixaban at a dose of 2.5 mg was administered twice during the 12–24-h period postoperatively. Patients were encouraged to initiate exercise from the first day after surgery as a prophylaxis measure for deep vein thrombosis. Patients were closely monitored to record the occurrence of hematoma and divided into a hematoma group and a non-hematoma group. All patients were followed up for at least 2 months, and no cases of symptomatic VTE were observed.

### Blood measurements

Peripheral blood was collected from each patient preoperatively and on postoperative days 1, 3, 7, and 14 for blood measurements. Blood coagulation functions were evaluated based on activated partial thromboplastin time (APTT), prothrombin time (PT), thrombin time (TT), and fibrinogen (FIB) using an automated blood coagulation analyzer (STAGO, France). Hemorheological parameters were measured using a hemorheological analyzer (LBY-N6C, Puli Inc., Beijing, China).

### Statistical analysis

Continuous data are presented as mean ± standard deviation values and were compared using Student *t* tests or one-way analysis of variance. Categorical data are presented as frequencies or percentages and were compared using *χ*^2^ tests. All statistical analyses were performed using SPSS 16.0 software for Windows (SPSS Inc., Chicago, IL, USA). *P* < 0.05 was considered statistically significant.

## Results

### Patient information

No significant differences were found in terms of sex, age, body mass index (BMI), and presence of diabetes or hypertension between patients in the hematoma group (*n* = 32) and those in the non-hematoma group (*n* = 114; Table 1). However, a significantly higher percentage of patients in the hematoma group had joint deformity (100 vs. 66.7 %, *P* < 0.001), and accordingly, the operation time was significantly longer for these patients (83.9 ± 3.1 vs. 70.5 ± 4.2 min, *P* < 0.001) than for patients in the non-hematoma group.

**Table 1:** Comparison of baseline data between patients in the hematoma group and the non-hematoma group

	Hematoma group (*n* = 32)	Non-hematoma group (*n* = 114)	*P* value
Sex (male/female)	11/21	40/74	0.94
Age (years)	70.6 ± 4.1	70.9 ± 4.7	0.932
Diabetes, *n* (%)	12 (37.5 %)	28 (24.6 %)	0.147
Hypertension, *n* (%)	25 (78.1 %)	71 (62.3 %)	0.095
BMI (kg/m^2^)	25 ± 2.2	26 ± 2.4	0.061
Joint deformity, *n* (%)	32 (100 %)	76 (66.7 %)	<0.001
Operation time (min)	83.9 ± 3.1	70.5 ± 4.2	<0.001

### Comparison of coagulation functions

**Fig. 1:**
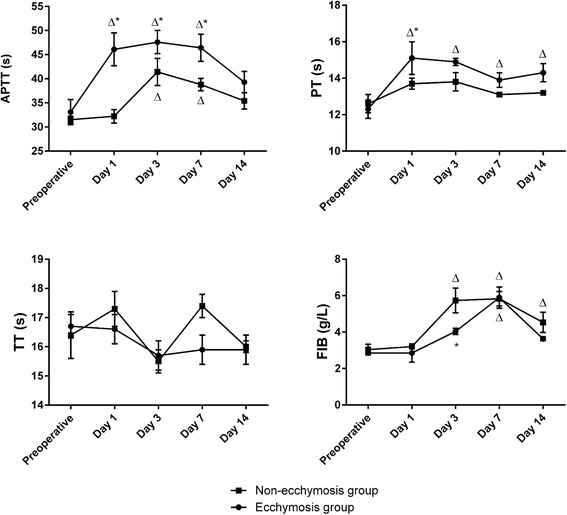
Changes in APTT, PT, TT, and FIB in the hematoma group and the non-hematoma group. *Triangle P* < 0.05 vs. preoperative values, *asterisk P* < 0.05 vs. the other group at the same time point

No significant differences were observed in APTT, PT, TT, and FIB between patients in the hematoma and non-hematoma groups preoperatively (*P* > 0.05). In comparison to preoperative values, APTT was significantly increased in the hematoma group on postoperative days 1, 3, and 7 and in the non-hematoma group on postoperative days 3 and 7 (*P* < 0.05, Fig. 1). In addition, the hematoma group showed a significantly higher APTT than the non-hematoma group on postoperative days 1, 3, and 7 (*P* < 0.05). Also, in comparison to preoperative values, PT was significantly increased in the hematoma group on postoperative days 1, 3, 7, and 14 (*P* < 0.05) but not in the non-hematoma group. In addition, FIB was significantly increased in the non-hematoma group on postoperative days 3, 7, and 14 but only on postoperative day 7 in the hematoma group (*P* < 0.05).

### Comparison of hemorheological parameters

**Fig. 2:**
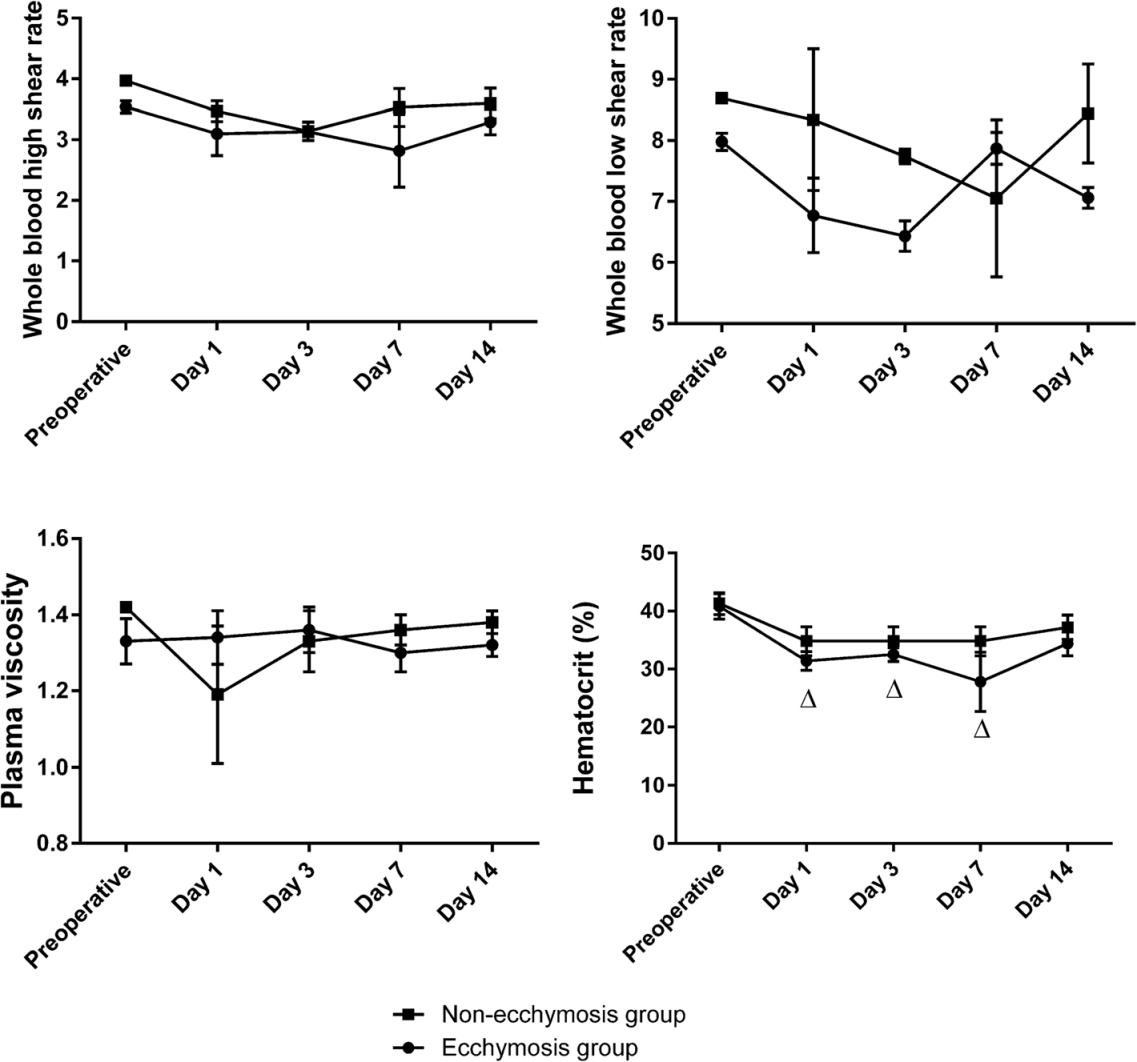
Changes in whole blood high shear rate, whole blood low shear rate, plasma viscosity, and hematocrit in the hematoma group and the non-hematoma group. *Triangle P* < 0.05 vs. preoperative values, *asterisk P* < 0.05 vs. the other group at the same time point

The whole blood high shear rates, whole blood low shear rates, plasma viscosities, and hematocrit levels did not differ significantly between the hematoma and non-hematoma groups at baseline or from postoperative days 1–14 (*P* > 0.05). However, the hematocrit level was significantly decreased on postoperative days 1, 3, and 7 in the hematoma group compared with the baseline value (*P* < 0.05, Fig. 2).

## Discussion

The specific mechanisms underlying the formation of ecchymoses after TKA remain unclear. However, our study showed that patients who experienced ecchymoses had significantly prolonged APTTs and PTs as well as significantly decreased hematocrit levels after TKA in comparison with baseline values. These findings may help clinicians identify patients at increased risk for hematoma formation after TKA.

In our study, preoperative coagulation functions did not differ significantly between patients with and without postoperative hematoma. Therefore, we are unable to predict the occurrence of hematoma based on preoperative coagulation functions. However, among patients with a hematoma, APTT was significantly prolonged to beyond 10 s on postoperative day 1 in comparison with baseline data. On the contrary, APTT in the non-hematoma group on postoperative day 1 was not significantly different from the baseline value. These results suggest that a prolonged APTT on postoperative day 1 may be predictive of hematoma formation. In addition, blood tests were performed on postoperative day 1 before the administration of apixaban. Therefore, the prolonged APTT was not caused by apixaban. Similar patterns were also observed for PT, which was significantly prolonged during the postoperative period in the hematoma group but not in the non-hematoma group. Thus, our results indicate that, together, prolonged APTT and PT on postoperative day 1 can be used to predict hematoma formation after TKA.

Hemorheological parameters reflect the properties of blood flow, with increased hemorheological parameters indicating higher blood viscosity and greater risks of thrombosis formation [14]. Blood viscosity is determined by both the whole blood viscosity and plasma viscosity. An increase in whole blood viscosity with a high shear rate can lead to decreased erythrocyte deformability, whereas an increase in whole blood viscosity with a low shear rate can lead to increased erythrocyte aggregation [15]. Our study found that the whole blood high shear rate, whole blood low shear rate, plasma viscosity, and hematocrit level did not differ significantly between patients in the hematoma and non-hematoma groups at baseline and from postoperative days 1–14. These results suggest that the erythrocyte deformability and aggregation were not significantly altered by the TKA procedure. We speculate that the use of apixaban helped to maintain the blood viscosity in the patients included in this study. A decreased hematocrit level is a sign of anemia or hemodilution [16], and in our study, hematocrit levels were significantly decreased in the hematoma group on postoperative days 1, 3, and 7 compared to the baseline value. In contrast, hematocrit levels in the non-hematoma group did not change significantly. We speculate that the hematoma group had a higher incidence of hemodilution, leading to decreased levels of coagulant factors and increased bleeding risk.

## Conclusions

In conclusion, prolonged APTT and PT on the first day after TKA together with a decreased hematocrit level may indicate an increased risk of hematoma formation. Postoperative use of apixaban may promote the formation of ecchymoses but is not a major contributing factor. Infusion of plasma or coagulant factors might be an effective prophylaxis measure to prevent hematoma formation after TKA.
